# Removal of selected pharmaceuticals from wastewater using spawn-based pellets of *Pleurotus ostreatus*: efficiency and environmental implications

**DOI:** 10.1186/s40694-026-00221-3

**Published:** 2026-09-10

**Authors:** Malin Hultberg, Elena Surra, Oksana Golovko

**Affiliations:** 1 Department of Biosystems and Technology, Swedish University of Agricultural Sciences (SLU), SE-234 56 Alnarp, Sweden; 2 Instituto Superior de Engenharia do Porto, Instituto Politécnico do Porto, REQUIMTE/LAQV, Rua Dr. António Bernardino de Almeida, 4249-015 Porto, Portugal; 3 Ed. Da Quinta da Lameira, Sines Tecnopolo, ZIL II, Lote 122A, 7520-309 Sines, Portugal; 4 Department of Aquatic Sciences and Assessment, Swedish University of Agricultural Sciences (SLU), SE-75007 Uppsala, Sweden

**Keywords:** Laccase, Life cycle assessment (LCA), Mycoremediation, Antibiotics, White-rot fungi

## Abstract

**Background:**

Pharmaceutical residues are increasingly detected in aquatic environments, and conventional wastewater treatment plants often provide insufficient removal. In this study, we evaluated the capacity of spawn-based pellets of the white-rot fungus *Pleurotus ostreatus* to remove 11 pharmaceuticals from synthetic wastewater and the secondary effluent from a municipal wastewater treatment plant and conducted a preliminary life cycle assessment (LCA) of the treatment process.

**Result:**

In synthetic wastewater, living fungal pellets reduced the pharmaceutical concentration by > 30% within 10 min compared to the control. Significant removal of nine compounds, particularly macrolide and sulfonamide antibiotics, was observed. Biosorption contributed to removal but accounted for only a minor fraction. In the secondary effluent, total pharmaceutical concentrations decreased by 51–66% after 2–24 h, with time-dependent removal observed for several compounds. LCA results indicated that fungal treatment combined with incineration of spent biomass had lower environmental impacts than landfilling, largely due to energy recovery. The major contributors to environmental burden were energy demands, particularly due to sterilisation during grain spawn preparation and aeration.

**Conclusion:**

Use of spawn-based pellets of *P. ostreatus* is a biologically based treatment technology for pharmaceutical removal. Future work should address transformation products, toxicity, and process optimization to improve sustainability at scale.

**Supplementary Information:**

The online version contains supplementary material available at https://doi.org/10.1186/s40694-026-00221-3.

## Introduction

Good-quality water is essential for all forms life, yet this resource is increasingly threatened by climate change, rapid population growth, and the intensive use of chemicals. Conventional wastewater treatment plants (WWTPs) efficiently remove nutrients such as nitrogen and phosphorus, as well as readily degradable organic carbon. However, their removal efficiency for organic micropollutants, including pharmaceuticals, varies widely and depends on the physicochemical properties of the compounds and the treatment processes applied [1]. Consequently, substantial research efforts are focused on developing and optimizing alternative treatment technologies capable of effectively removing organic pollutants from aquatic environments. In this context, bioremediation strategies based on oxidoreductase enzymes, including laccases and peroxidases, have gained increasing attention, as reflected by numerous recent review articles [2–5].

Oxidoreductase enzymes are produced by various organisms, including plants, fungi, and bacteria [5]. Among these, white-rot fungi are particularly well known for their role in terrestrial ecosystems as highly efficient degraders of lignocellulosic material. They secrete large quantities of ligninolytic enzymes such as laccases and peroxidases during growth and the subsequent breakdown of plant biomass. These enzymes exhibit broad substrate ranges, and their catalytic action is closely linked to the generation of hydrogen peroxide and free radicals, which facilitate the degradation of complex molecules [6]. As a result, the ligninolytic enzymes can act on a wide variety of compounds beyond lignin. Notably, both the fungi and their extracellular enzymes have been shown to degrade a broad spectrum of pharmaceuticals and other organic pollutants in aquatic environments [5, 7]. Considering the use of living fungi, removal mechanisms involve both biosorption to fungal biomass—potentially followed by intracellular degradation via systems such as cytochrome P450—as well as direct degradation by extracellular enzymes [8].

A variety of treatment technologies have been evaluated for their ability to remove pharmaceuticals, including UV irradiation, adsorption, membrane separation, advanced oxidation processes, and emerging catalyst-based methods [9]. Many of these techniques achieve high removal efficiencies. However, life cycle assessment (LCA) studies of quaternary treatment processes indicate that these methods can introduce substantial environmental burdens [10]. Selecting an appropriate treatment technology is therefore crucial to minimizing indirect environmental impacts. In this context, biologically based approaches represent a promising alternative. This study therefore aimed to evaluate the ability of a recently developed technique using spawn-based pellets of the white-rot fungus *Pleurotus ostreatus* [11]. Use of *P. ostreatus* and its enzymatic system in bioremediation processes is well established [2, 5, 7] and this species has been highlighted as interesting in development in sustainable biobased technologies [12]. This type of spawn-based fungal pellet has previously been shown successful in removing pharmaceuticals from wastewater with a focus on pharmaceutical groups [13]. The present study concentrates on 11 specific pharmaceuticals and explores sorption to the pellets and removal from the water phase. Additionally, a preliminary LCA was performed to assess environmental impacts associated with this applied fungal-based treatment process. Thus, this study explores an applied fungal-based approach for treatment of wastewater while also integrating experimental data with a preliminary LCA. This provides novel information about contributions of key processes to the environmental impact of the treatment and provide a foundation for its further development.

## Materials and methods

### Microorganism and pellet production

**Fig. 1:**
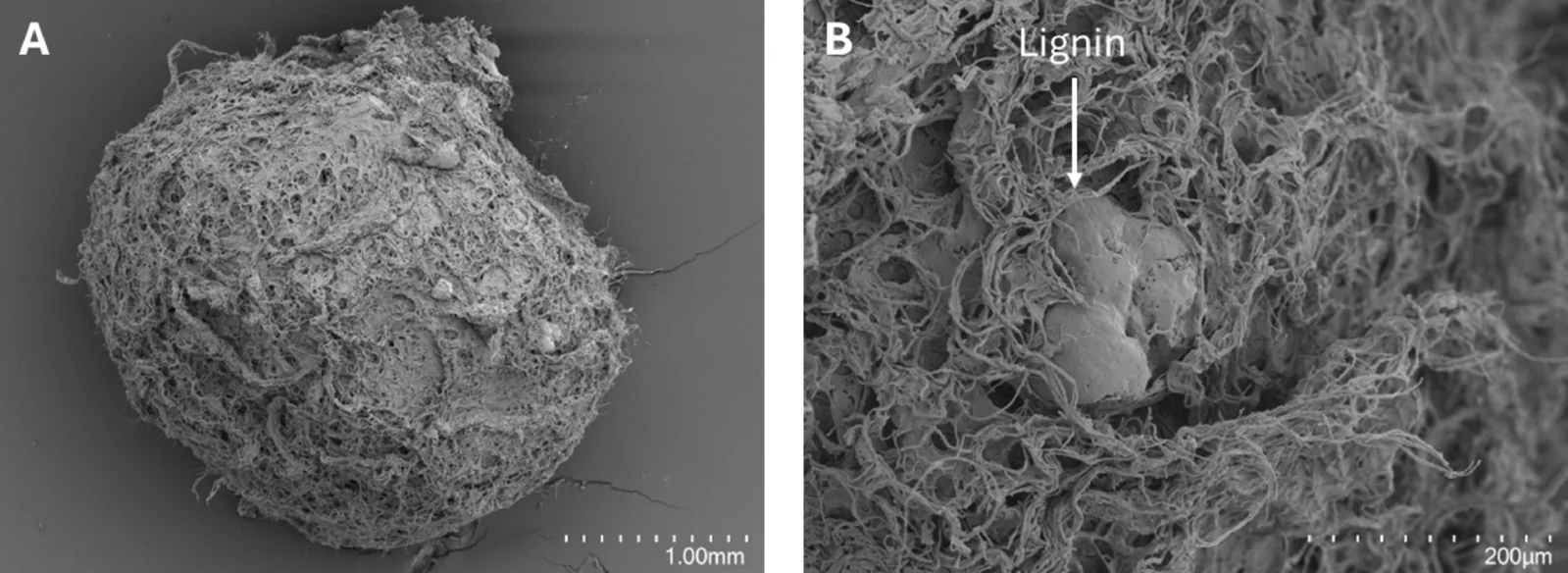
The spawn-based pellets are composed of grain and the hypha growing out of the grain (**A**). Particles of kraft lignin, added to induce production of ligninolytic enzymes, are entrapped in the mycelium (**B**)

Grain spawn of the white-rot fungal species *Pleurotus ostreatus* M2191 was obtained from Mycelia BVBA, Belgium. Fungal pellets were produced using a concentration of 40 g/L of grain spawn (fresh weight) and kraft lignin (Sigma-Aldrich 370959), added at start, in a concentration of 4 g/L. A previous study has confirmed that laccase is the major ligninolytic enzyme produced by the strain *P. ostreatus* M2191 under the used conditions and that enzyme activity reaches a maximum between day 3–5 and then declines [11]. The capacity of this type of fungal pellets to reduce different pharmaceutical groups in various wastewaters has been confirmed [13]. Laccase activity in the samples was quantified colorimetrically by monitoring the oxidation of 2,6-dimethoxyphenol (DMP), as described by Parenti et al. [14]. The pellet is composed of grain and fungal hyphae, growing out from the grain, and lignin and has an approximate size of 0.5–2 cm (Fig. 1). The pellets were imaged using a scanning electron microscope (SEM; Hitachi SU3500) at 5 kV. Gold coating and SEM imagining was performed at the Microscopy Platform, Lund University, Sweden.

### Experimental set-up

*Experiment 1: Synthetic Wastewater.* The experiments were carried out in 300-mL Erlenmeyer flasks (borosilicate glass) placed on a horizontal orbital shaker (VWR Advanced 5000 Shaker, Radnor, PA, USA) operated at 100 rpm. Incubations were performed at ambient laboratory temperature (20–22 °C). Each Erlenmeyer glass flask contained 50 ml of synthetic wastewater [15], grain spawn and kraft lignin in the concentrations described above. The inoculated flasks were cultivated for four days reaching a laccase activity of 425 ± 35 U/L. Autoclaved samples were used as the control treatment. The pellets retained their integrity after they were autoclaved. The absence of enzymatic activity was confirmed in the autoclaved glass flasks. Thus, the control was composed of pellets with dead fungal biomass and without laccase activity. The glass flasks were then spiked with a selected pharmaceuticals (described below) in a nominal concentration of 10 µg/L of each pharmaceutical. The samples in both control and treatment were subjected to 10 min of incubation on an orbital shaker (100 rpm) and then centrifuged at 3000 x g for 15 min to remove the fungal pellets. The rationale for exposing the wastewater to fungal pellets for only 10 min was to primarily investigate adsorption by the fungal matrix versus extracellular enzymatic degradation. However, pharmaceutical removal by metabolism-linked mechanisms cannot be excluded.

*Experiment 2: Municipal Wastewater.* Wastewater sample was collected from a municipal WWTP treating 200 000 population equivalents (PE). The treatment steps at the WWTP included an initial separation step, primary clarification, activated sludge, secondary clarification, and precipitation of phosphates with FeCl_3_ before the release to the catchment. The treated wastewater, referred to as secondary effluent, was collected in 1 L high-density polyethylene containers prewashed with methanol. The secondary effluent was transported cold, stored at 4 °C until use in the experiments, which were performed within one week and no pretreatment was applied to the wastewater before inoculation of the pellets. The fungal pellets were produced as described above using sterile distilled water, grain spawn and lignin. This pre-cultivation was performed to initiate fungal growth and laccase production. After 48 h of cultivation, the pellets were collected by coarse filtration (800 μm) and transferred to a corresponding volume of secondary effluent. The secondary effluent was sampled after 2 h, 6 h and 24 h of treatment to determine impact on the selected pharmaceuticals. The same environmental conditions as described for the first experiment were applied. For this experiment, wastewater controls without fungal pellets were analyzed immediately at 0 h to determine initial pharmaceutical concentrations and provide the untreated baseline for assessing fungal removal efficiency.

The liquid samples from both experiments were immediately stored in a freezer at -20 °C until analysis, which was performed within one week. The solid phase collected from experiment 1 was lyophilized and extracted as described below. All experiments were performed with three replicates.

### Chemicals and analysis

For chemical analysis, reference standards for 11 target pharmaceuticals (azithromycin, erythromycin, sulfamethoxazole, caffeine, fluconazole, tramadol, trimethoprim, metoprolol, lidocaine, venlafaxine, and desvenlafaxine) were purchased from Sigma-Aldrich (Sweden). The physicochemical properties of these compounds are presented in the Supplemental Information (Table S1). The target compounds were selected based on literature reports regarding their occurrence and ubiquity in aquatic environments, as well as their high levels of human use and consumption worldwide [16–18]. All analytical standards used were of high purity (> 95%).

Solid-phase samples were extracted using a validated in-house protocol based on Kodešová et al. [19]. Briefly, 1 g (dw) of each sample was placed in a 10 mL glass vial and extracted with 3 mL of solvent A (acetonitrile/water 1:1, 0.1% formic acid) by 15-min ultrasonication. The supernatant was filtered (0.2 μm regenerated cellulose). The residue was then extracted with 3 mL of solvent B (acetonitrile/2-propanol/water 3:3:4) under the same conditions, filtered, and combined with the first extract. A 1 mL aliquot was used for analysis. Blank samples were processed identically without adding sample material.

Water samples were filtered through 0.2 μm regenerated cellulose filters and 1 mL aliquots were used for analysis.

Analysis employed a Dionex UltiMate 3000 UHPLC system coupled to a TSQ Quantiva triple quadrupole mass spectrometer (Thermo Scientific). Separation used an Acquity UPLC BEH-C18 column (100 mm × 2.1 mm, 1.7 μm). Data processing was performed using Xcalibur and TraceFinder™.

### No target analytes were detected in blanks or control samples

The limit of quantification (LOQ) for solid samples ranged from 5 µg/kg for azithromycin to 12 µg/kg for metoprolol and sulfamethoxazole. For water samples, the LOQ ranged from 0.1 ng/L for caffeine and erythromycin to 0.45 ng/L for metoprolol. Absolute recovery ranged from 20% for azithromycin to 56% for both caffeine and lidocaine.

*Statistical analysis.* All experiments were conducted in triplicate for each treatment. Statistical analyses were performed using Minitab v.2020, with significance assessed at *p* < 0.05 using ANOVA, Tukey’s post hoc test, and Student’s t-test. Results are expressed as mean ± standard deviation (SD).

### Life cycle assessment

A Life Cycle Assessment (LCA) was conducted to evaluate the environmental impacts of the fungal pellet-based treatment process. The LCA was developed according to the ISO 14,040 and 14,044 standards and includes four steps: (i) Goal and Scope, (ii) Life Cycle Inventory (LCI), (iii) Impact Assessment and (iv) Interpretation of the results. The first two steps are described in this section, and the impact assessment and interpretation of the results are reported in the following sections.

*Goal and Scope.* This LCA analyses the environmental impacts generated by the fungal treatment of wastewater effluent, considering two different scenarios for the End-of-Life Stage of spent Biomass: Incineration (scenario 1) and Landfill (scenario 2). A volume of 1 L of simulated secondary effluent was chosen as a functional unit (F.U.), since it is consistent with the goal of the study and with the function of the system [20] and can be used to preliminarily compare the impacts with previously studied technologies.

This LCA was performed according to a “cradle-to-gate” approach within the system boundaries (Fig. S1). This analysis does not include capital goods. The allocation of the impacts was developed following the “Consequential” approach according to the “avoided-burden method”, as proposed by Clift et al. [21]. This LCA study was developed using the SimaPro 9.1.7 software package from PréConsultant, equipped with the Ecoinvent v.3.1 database, applying the ReCiPe2016 Midpoint H v.1.04 (ReCiPe) [22].

*Life Cycle Inventory.* Tables S2-S4 report the Life Cycle Inventory (LCI) used for modelling the fungal treatment under study. Tables S5-S7 report the assumptions, and the calculations made for development of the LCI. The LCI was developed including the production process for the grain spawn, according to the adapted procedure retrieved elsewhere [23], as well as pellet production and fungal treatment. During spawn production, electricity is consumed for the preparation of pure fungal inoculum, grain substrate processing, and heat generation for inoculum sterilization. In pellet production, electrical energy is required to operate the bioreactor for aeration and to provide heat for sterilization of both the medium and the reactor. During wastewater treatment, electricity is primarily used for pumping wastewater into the bioreactor and for operating aeration systems [24]. The inputs of electricity come from the Swedish grid [25].

## Result

### Pharmaceutical removal in synthetic wastewater (experiment 1)

**Fig. 2:**
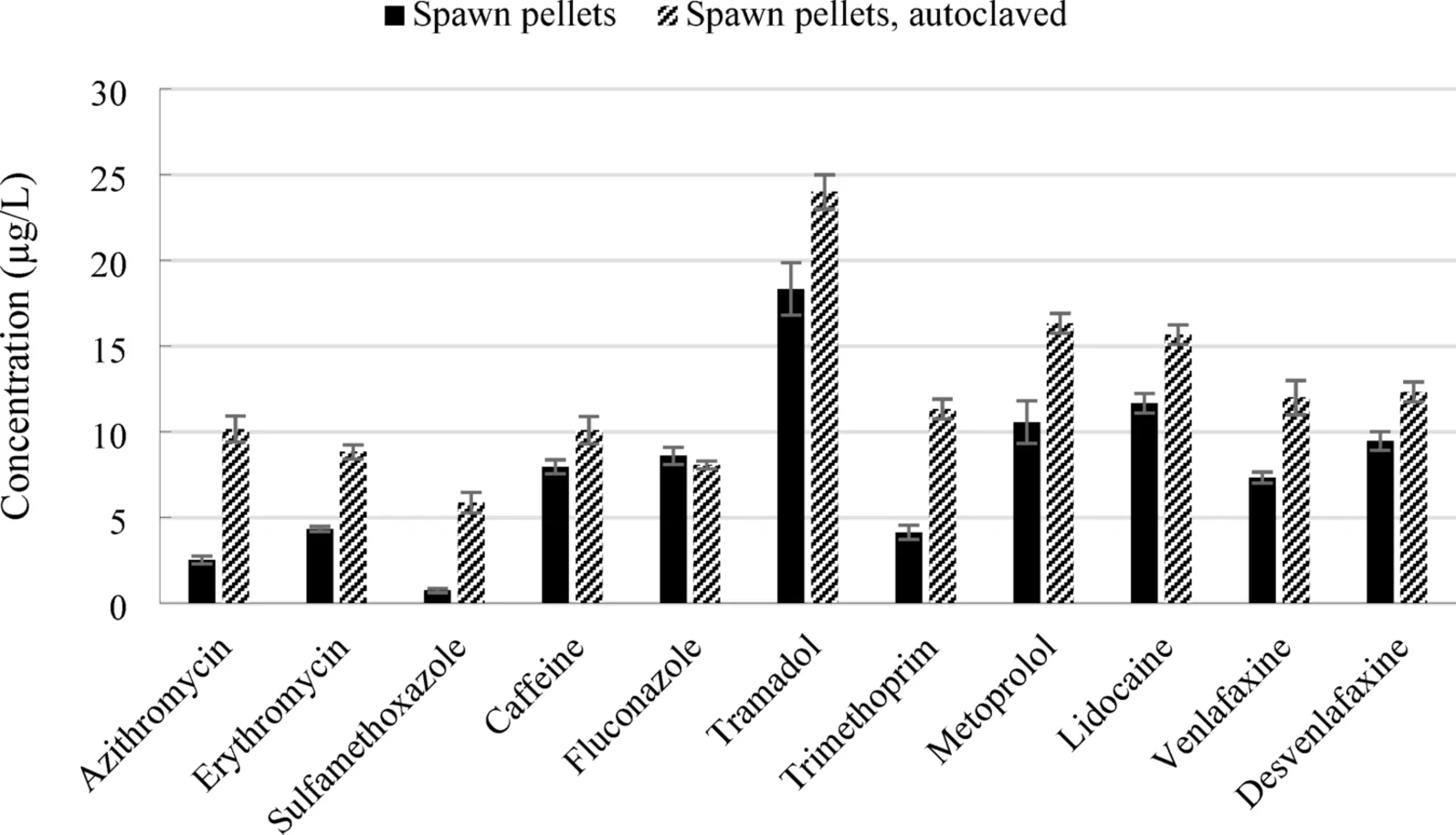
Concentration of pharmaceuticals in synthetic wastewater after 10 min of exposure to fungal pellets of *Pleurotus ostreatus*. Autoclaved pellets without laccase activity were used as a control. Mean ± std, *n* = 3

In experiment 1, a significant difference in the total sum of pharmaceuticals in the water phase was observed after treatment with living fungal pellets (85.7 ± 5.5 µg/L) compared with the autoclaved control (134.7 ± 5.5 µg/L). When evaluating the effect of treatment on individual target compounds, concentrations of the antibiotics azithromycin, erythromycin, sulfamethoxazole, and trimethoprim decreased by more than 50% relative to the control. Overall, of the 11 target compounds analyzed, nine showed significant reductions. For the concentration of caffeine and fluconazole no impact of treatment was observed (Fig. 2, Table S8).

**Fig. 3:**
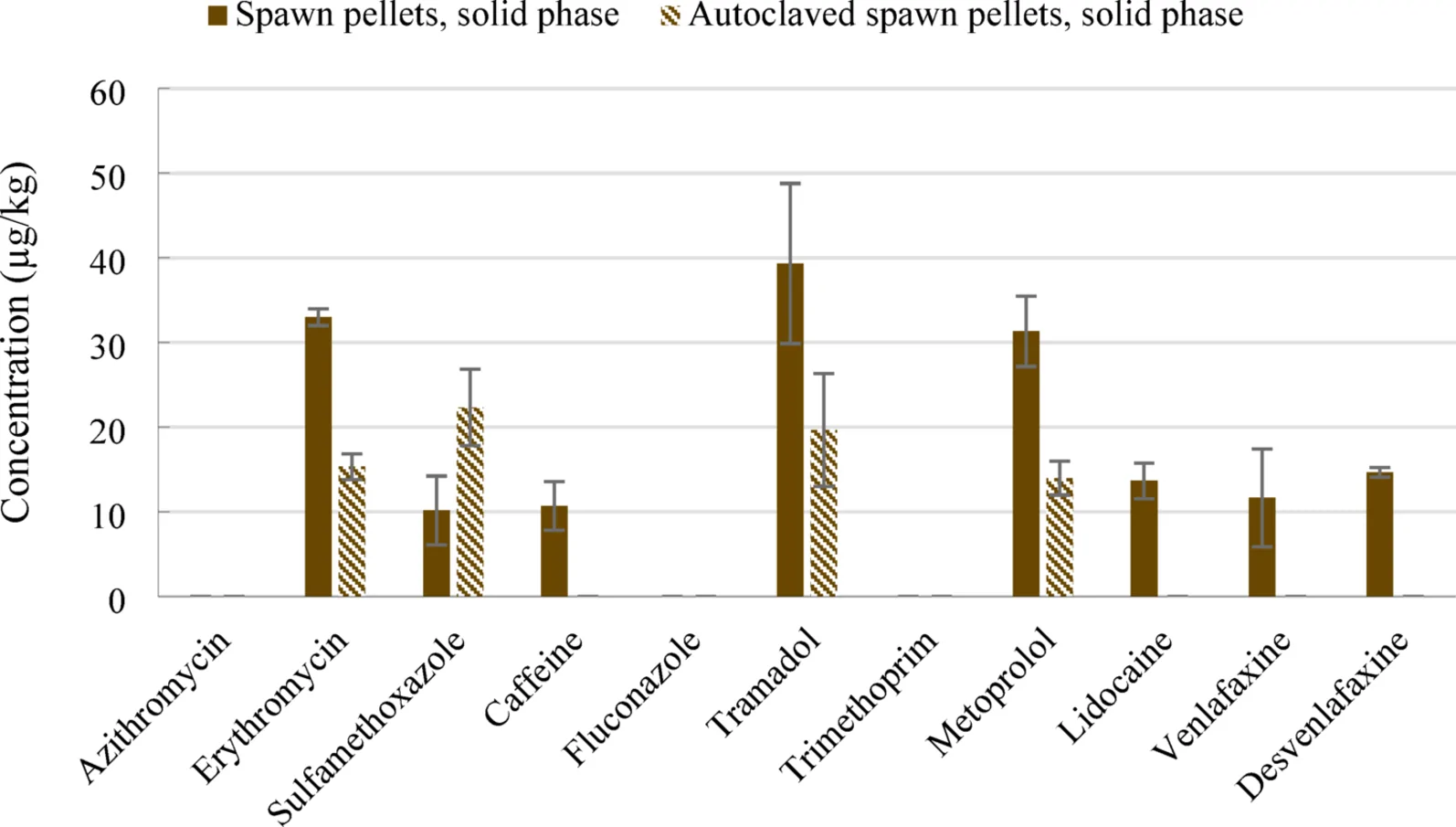
Concentration of pharmaceuticals in fungal pellets of *Pleurotus ostreatus*. Living and dead (autoclaved) fungal pellets were included in the experiment. Mean ± std, *n* = 3

The biosorption of the studied pharmaceuticals was significantly higher in pellets with living fungi (Fig. 3). Total sum of pharmaceuticals in the pellets was 71.3 ± 13.7 µg/kg dry weight (dw) in the autoclaved control and 165.5 ± 9.5 µg/kg dw in the treatment with living fungi. The amount of biomass in the treatment was 25.1 ± 0.8 g/L dw in the treatment with living fungus and 23.5 ± 0.1 g/L dw in the autoclaved treatment. Thus, in the treatment with living fungi, a total amount of approximately 4 µg/L of pharmaceuticals was removed by sorption to the pellets while approximately 2 µg/L was removed by sorption in the autoclaved treatment. Erythromycin, tramadol, and metoprolol exhibited the greatest tendency to accumulate in living pellets, whereas azithromycin, fluconazole, and trimethoprim were below the detection limit (Fig. 3). Sulfamethoxazole was the only compound detected in higher concentration in the autoclaved control than in the treatment with living fungus.

### Pharmaceutical removal in secondary effluent (experiment 2)

All targeted compounds, but azithromycin, was detected in the secondary effluent used in experiment 2 (Table 1). The concentration of the pharmaceuticals varied considerably with erythromycin having the lowest concentration, approximately 6 ng/L, while desvenlafaxine was detected in a concentration of almost 2 µg/L.

**Table 1:** The concentration of the studied pharmaceuticals in the secondary effluent used in experiment 2

Pharmaceuticals	Conc. (ng/L)
Azithromycin	BDL*
Erythromycin	5.6 ± 1.7
Sulfamethoxazole	676.7 ± 20.8
Caffeine	52.7 ± 5.9
Fluconazole	60.0 ± 5.6
Tramadol	273.3 ± 15.3
Trimethoprim	85.3 ± 4.0
Metoprolol	118.7 ± 22.0
Lidocaine	86.0 ± 6.1
Venlafaxine	416.7 ± 32.1
Desvenlafaxine	1833.0 ± 252.0
Total sum	3608.3 ± 237.4

Mean ± std is shown, *n* = 3

*BDL  below detection limit

**Fig. 4:**
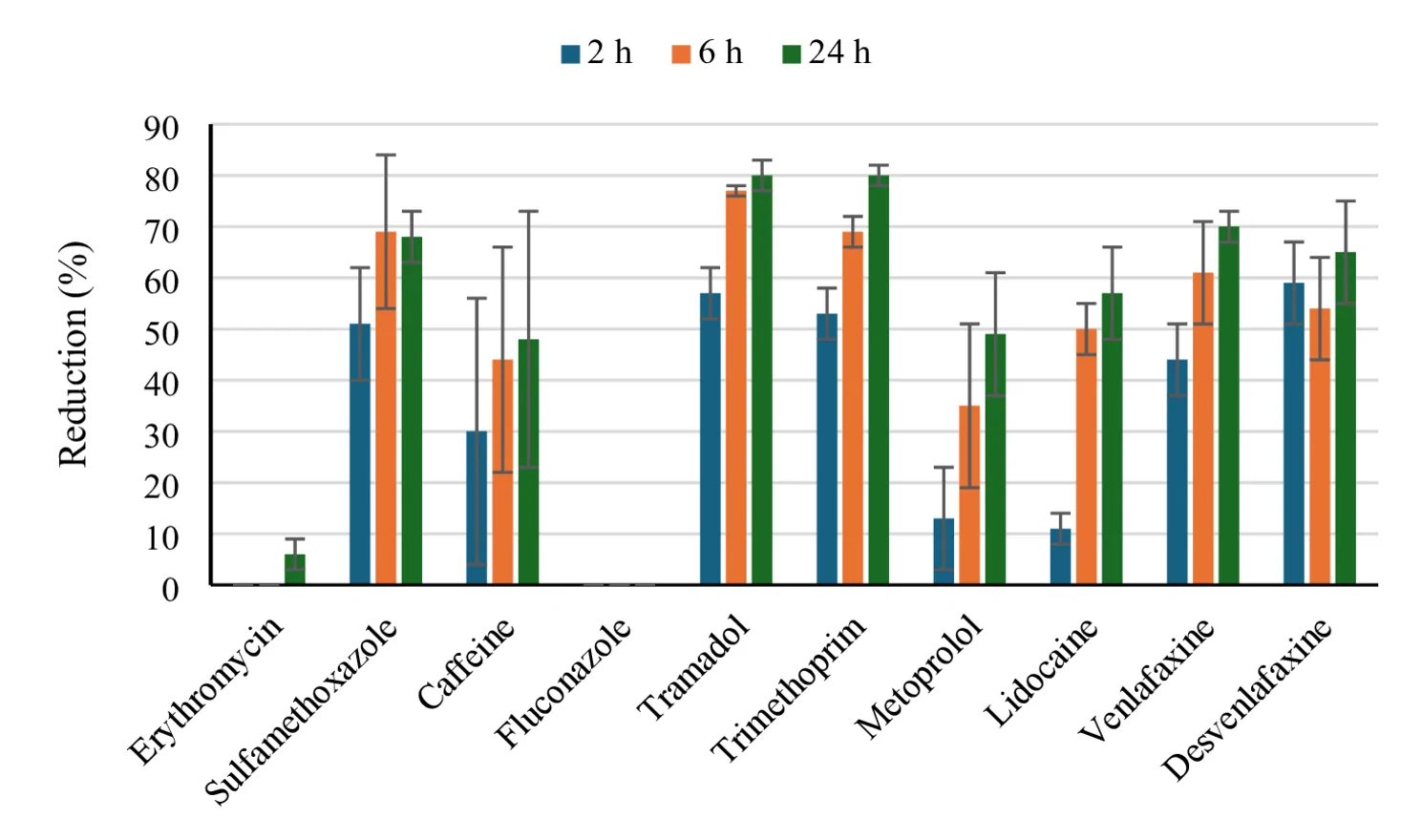
The relative reduction (%) of selected pharmaceuticals in the secondary effluent after treatment with fungal pellets of *P. ostreatus*. Mean ± std, *n* = 3

Treatment with fungal pellets resulted in a significant reduction of 51 ± 4% (2 h treatment), 58 ± 7% (6 h treatment) and 66 ± 4% (24 h treatment) compared to the initial concentration. There was no significant difference regarding the treatment time considering total sum. All compounds but erythromycin and fluconazole were significantly reduced by treatment (Fig. 4, Table S8). For the individual compounds, the impact of treatment time varied. Tramadol, trimethoprim, metoprolol, lidocaine and venlafaxine showed a significant increase in degradation with increasing treatment time while no significant difference was observed for the others.

### Life cycle assessment

Table 2 shows the results of the total environmental impact (ReCiPe) associated with fungal treatment. Fungal treatment with final incineration of the spent biomass (Scenario 1) is more environmentally advantageous in most of the categories of impact analysed than that in which the spent biomass is landfilled (Scenario 2). The percentages of reduction in the case of incineration were 103% in the Global Warming category of impact, 43% in Fossil Resource Scarcity, and 49% in Mineral Resource Scarcity, with the highest observed in the category of impact of ozone formation (> 160%). This result is mainly due to the heat recovery associated with the incineration process of biowaste, which can cut-off the emissions associated with the combustion process, and avoid the emission of greenhouse gases (GHG) produced by biowaste fermentation during landfilling. Analysis of the processes contribution showed that the advantages of scenario 1 (incineration) are associated with the heat recovery for electric energy production. It should also be highlighted that the production process of grain spawn represents the main contributor to the final values of impact in the Ecotoxicity and Human Toxicity-related categories of impact.

**Table 2:** Total environmental impacts of fungal treatment (ReCiPe method) of Scenario 1 (Incineration) and Scenario 2 (Landfill)

Impact category	Unit	Scenario 1 (incineration)	Scenario 2 (landfill)	Decrease (%) (Sc1 vs. Sc2)
Fine particulate matter formation	kg PM_2.5_ eq	-1.50E-04	-1.42E-04	6%
Fossil resource scarcity	kg oil eq	1.91E-03	3.33E-03	43%
Freshwater ecotoxicity	kg 1.4 DCB eq	1.77E-03	1.77E-03	0%
Freshwater eutrophication	kg P eq	-6.03E-05	-5.47E-05	10%
Global warming	kg CO_2_	-9.80E-04	3.02E-02	103%
Human carcinogenic toxicity	kg 1.4 DCB eq	-2.70E-03	-2.70E-03	0%
Human non-carcinogenic toxicity	kg 1.4 DCB eq	1.64E-01	1.64E-01	0%
Ionizing radiation	kBq Co-60 eq	4.82E-03	4.99E-03	3%
Land use	m^2^a crop eq	5.98E-02	5.98E-02	0%
Marine ecotoxicity	1.4 DCB eq	1.92E-03	1.92E-03	0%
Marine eutrophication	kg N eq	1.84E-04	2.00E-04	8%
Mineral resource scarcity	kg Cu eq	3.98E-05	7.82E-05	49%
Ozone formation, Human health	kg NOx eq	-2.00E-05	2.21E-05	190%
Ozone formation, Terrestrial ecosystems	kg NOx eq	-1.73E-05	2.49E-05	169%
Stratospheric ozone depletion	kg CFC11 eq	2.99E-07	3.10E-07	4%
Terrestrial acidification	kg SO_2_ eq	2.92E-04	3.18E-04	8%
Terrestrial ecotoxicity	1.4 DCB eq	1.99E-01	1.99E-01	0%
Water consumption	m^3^	1.17E-03	1.33E-03	12%

All values are referred to F.U. (1 L simulated wastewater)

## Discussion

In experiment 1, fungal pellets were studied for its capacity to remove 11 pharmaceuticals from synthetic wastewater and their sorption to the pellets. Treatment with living pellets resulted in significantly lower concentration of pharmaceuticals in the water phase in comparison to a control based on dead fungal biomass and without laccase activity. Sorption to the fungal pellets were studied and accumulation of pharmaceuticals was significantly higher with living fungi compared to the autoclaved control. However, the accumulation of pharmaceuticals in the pellet was minor, compared to the concentration of pharmaceuticals in the water phase. Thus, the extracellular enzymes produced by the fungal pellets are important factors in the removal process. Alongside extracellular enzymatic degradation and biosorption, also metabolism-linked mechanisms (e.g., intracellular transformation) may have contributed to higher pharmaceutical removal with the living fungal pellets. Still, experiment 1 was performed with a short time of exposure to the fungal pellets (10 min) and the contribution of metabolism-linked mechanisms was most likely low.

In studies of biosorption of pollutants in water by fungi, much work has been focused on the use of dead fungal biomass, mainly due to practical advantages in handling of the biomass [26]. For biosorption of dyes, dead fungal biomass has often performed better than living fungi and this has been explained by heat-induced modifications of the cell wall [27]. On the other hand, a study comparing living and dead fungal biomass in their removal of a wide spectrum of pharmaceuticals observed considerable differences regarding the individual pharmaceutical and no advantage in using autoclaved fungal biomass [28]. The observed differences in sorption behaviour between living and dead fungal pellets are influenced by pH-dependent ionization of cell-wall functional groups (e.g., carboxylate/phosphate), which typically yields a net negative surface charge near neutral pH and modulates the electrostatic interaction with the compound [29]. Due to the sorption of pharmaceuticals to the pellets, they should be removed after water treatment and disposed of. This is not a major constrain as the size of the pellets allows for harvesting by coarse filtration. In terms of biomass harvesting, the use of filamentous fungi has clear benefits compared to treatment based on microalgae or bacteria.

In experiment 2, using secondary effluent from wastewater, a significant reduction of total concentration of pharmaceuticals was observed after treatment with fungal pellets. While no significant effect of extending the treatment time was observed considering total sum of pharmaceuticals, several of targeted pharmaceuticals showed increased removal over time. This is a somewhat contradictory result, which can be explained by the behaviour of compound desvenlafaxine. This antidepressant was detected in very high concentration in the wastewater compared to the other compounds and no impact of extended treatment time was observed in its degradation. Thus, desvenlafaxine dominate total concentration trends and masked time-dependent effects.

In the first experiment, a high reduction in the antibiotics azithromycin, erythromycin, sulfamethoxazole and trimethoprim was observed. Similarly, Mayans et al. [30] reported high removal rates of sulfamethoxazole and trimethoprim after exposure to *P. ostreatus*, where Alokpa et al. [31] reported some degradation of azithromycin, but considerably less than that of sulfamethoxazole and trimethoprim, after exposure to a laccase from the white-rot fungus *Trametes versicolor*. The high removal of sulfamethoxazole and trimethoprim, ranging between 70 and 80% compared to initial concentration, was confirmed in the second experiment where the fungal pellet was applied in real wastewater. However, the capacity for removal of azithromycin and erythromycin could not be confirmed as azithromycin was not detected in the sampled wastewater and no impact on the concentration of erythromycin was observed. Notably, erythromycin was detected in a very low concentration in the municipal wastewater, and this might be a factor explaining its low removal. One target compound, fluconazole, was unaffected by treatment in both experiments. This compound belongs to the commonly used azole fungicides, whose presence in the environment is a concern because of low biodegradation, although photolysis and Fenton reactions have been demonstrated to increase their degradation [32]. Differences in removal of individual pharmaceuticals removal can be explained by compound-specific physicochemical properties. Aromatic compounds are generally more susceptible to fungal degradation compared to non-aromatic compounds [33, 34]. Laccase-mediated oxidation is favoured by electron-donating substituents and inhibited by electron-withdrawing groups, which may further explain variability among antibiotics. Additionally, predominantly anionic compounds under neutral pH conditions may experience reduced removal due to electrostatic repulsion from the negatively charged fungal surface [29].

Overall, the spawn-based fungal pellets evaluated in this study clearly demonstrated their potential to impact pharmaceutical concentration in water. This is of interest as the use of enzyme-mediated processes to degrade and eliminate pollutants from contaminated water is an area of intensive research [2–5]. Still, there is a need to develop strategies and processes for the efficient application of these enzymes [7]. Recent studies have suggested enzyme immobilization on carriers such as nanofibrous membranes or hydrogels as a way forward [35, 36]. This is, however, technically advanced and costly techniques and as discussed by Pereira et al. [37] direct use of living white-rot fungi which can be autoimmobilized in the form of pellets [38] should also be considered. Thus, the spawn-based fungal pellet could potentially be useful for obtaining in-situ production of laccase in water. A preliminary assessment of the environmental impact associated with fungal pellet treatment and based on LCA was therefore performed. The results highlight that the electric energy consumption required for the preparation (boiling and sterilization) of the grain to obtain grain spawn represents the main contributor in the most significant categories of impact, followed by the electric energy consumption required for aeration during pellet production. This result, which identifies the electricity used to sterilize the media to produce fungal biomass as the highest contributor in nearly all categories of impact, agrees with the literature [24]. Future research efforts in process development should focus on the optimizing the production of the grain spawn, for example exploring less energy demanding pretreatments of the grain before inoculation of the fungus.

The values of environmental impact obtained in this work, are generally one order of magnitude higher than those observed at larger scales for these more consolidated technologies [39]. The difference mainly related to the specific electric energy consumption, which in the case of fungal treatment explored in this study is associated with the grain spawn production and aeration of the reactor. This result was not unexpected, larger-scale processes are characterized by lower energy consumption than laboratory scale processes. This is due to the increase in efficiency in the large-scale process and suggests a margin of improvement for the fungal treatment which, however, remains to be proven. The final biomass valorisation for heat production (incineration in Scenario 1) allows us to obtain some credits in energy related categories of impact and has always to be preferred to landfills. Composting can also be an alternative, however, as sorption of pharmaceuticals to the fungal biomass was observed, inclusion of the harvested biomass in a biochar process is potentially a better option to explore.

Enzymatic degradation of pharmaceuticals raises the issue of degradation products, and this aspect was not explored in this study. Treatment with white-rot fungi has often been reported to transform complex environmental pollutants into less toxic or non-toxic compounds [40]. Still, a recent study focusing on fungal degradation of organophosphate flame retardant reported increased toxicity after treatment [41]. Thus, no general conclusions can be drawn on the impact on toxicity after treatment with white-rot fungi. Therefore, parallel with development of the spawn-based pellets from technical and economical perspectives, the impact of this treatment on toxicity needs to be further explored. Additionally, a risk factor that needs to be addressed is the effect of high laccase activity in aquatic ecosystems. The need for studies on this topic has been pointed out previously by Gasser et al. [42], but to the best of our knowledge such studies are still lacking.

## Conclusion

In our previously published work [13], upon which the present manuscript was built, spawn-based pellets of white-rot fungi showed promising potential for removing different groups of pharmaceuticals from wastewater. The present study concentrates on 11 specific compound and shows that the living fungi clearly outperform autoclaved biomass. Short-time treatment resulted in a substantial reduction of pharmaceutical concentration in synthetic wastewater and significant decreases for nine out of eleven target compounds. In particular, the antibiotics azithromycin, erythromycin, sulfamethoxazole, and trimethoprim were reduced by more than 50%. The potential for reduction of antibiotics by the fungal treatment were confirmed in secondary effluent collected from a municipal wastewater treatment plant. The lack of removal observed for caffeine and fluconazole highlights the compound-specific nature of fungal treatment and points to limitations related to physicochemical properties. Biosorption contributed to pharmaceutical removal in both living and autoclaved pellets; however, accumulation was significantly higher in living biomass. The concentration of pharmaceuticals retained in the biomass was small relative to the aqueous concentrations, demonstrating that sorption alone cannot explain the observed removal and highlights the importance of enzymatic degradation. A preliminary LCA was performed to assess environmental impacts associated with the use of spawn-based fungal pellets. The major contributors to environmental burden were energy demands, particularly due to sterilisation during grain spawn preparation, with this information providing a foundation for further development of the technique. The presence of sorbed compounds on the fungal pellets suggests a need for post-treatment of the harvested biomass and appropriate disposal. This is in line with the results from the LCA which suggests incineration of the spent biomass as advantageous compared to landfilling.

## Supplementary Information

Below is the link to the electronic supplementary material.

Supplementary Material 1.

## Data Availability

The data will be made available upon request.
