# Supplementary material for: Removal of selected pharmaceuticals from wastewater using spawn-based pellets of Pleurotus ostreatus: efficiency and environmental implications
Source: Fungal Biol Biotechnol. 2026 Sep 10;13:16. doi: 10.1186/s40694-026-00221-3 (PMC13560132; doi:10.1186/s40694-026-00221-3)
Supplement: Supplementary file 1 [file 40694_2026_221_MOESM1_ESM.docx]

**Supplemental information**

**Title: Removal of selected pharmaceuticals from wastewater using spawn-based pellets of *P. ostreatus*: Efficiency and environmental implications**

Authors: Malin Hultberg^a,*^, Elena Surra^b,c^, Oksana Golovko^d^

^a^Department of Biosystems and Technology, Swedish University of Agricultural Sciences (SLU), SE-234 56 Alnarp, Sweden

^b^REQUIMTE/LAQV, Instituto Superior de Engenharia do Porto, Instituto Politécnico do Porto, Rua Dr. António Bernardino de Almeida, 4249-015 Porto, Portugal

^c^Ed. Da Quinta da Lameira, Sines Tecnopolo, ZIL II, Lote 122A, 7520-309 Sines, Portugal

^d^Department of Aquatic Sciences and Assessment, Swedish University of Agricultural Sciences (SLU), SE-75007 Uppsala, Sweden

| Compound | Category | Type | CAS number | Molecular formula | Monoisotopic | pKa | logKow |
| --- | --- | --- | --- | --- | --- | --- | --- |
|  |  |  |  |  | mass (Da) |  |  |
| Azithromycin | Pharmaceutical | Antibiotic | 83905-01-5 | C38H72N2O12 | 748.5 | 8.7 | 4.02 |
| Caffeine | Stimulant |  | 58-08-02 | C8H10N4O2 | 194.1 | 10.4 | -0.07 |
| Desvenlafaxine | Pharmaceutical | Antidepressant | 93413-62-8 | C16H25NO2 | 263.1 | 9.18 | 2.72 |
| Erytromycin | Pharmaceutical | Antibiotic | 114-07-8 | C37H67NO13 | 733.4 | 8.8 | 3.06 |
| Fluconazole | Pharmaceutical | Antifungal | 86386-73-4 | C13H12F2N6O | 306.1 | 2.03 | 0.25 |
| Lidocaine | Pharmaceutical | Anesthetic | 137-58-6 | C14H22N2O | 234.1 | 8 | 1.66 |
| Metoprolol | Pharmaceutical | Beta blocker | 51384-51-1 | C15H25NO3 | 267.1 | 9.7 | 1.69 |
| Sulfamethoxazole | Pharmaceutical | Antibiotic | 723-46-6 | C10H11N3O3S | 253.0 | 2 | 0.48 |
| Tramadol | Pharmaceutical | Analgesics (painkiller) | 27203-92-5 | C16H25NO2 | 263.1 | 9.4 | 3.01 |
| Trimethoprim | Pharmaceutical | Antibiotic | 738-70-5 | C14H18N4O3 | 290.1 | 7.1 | 0.73 |
| Venlafaxine | Pharmaceutical | Antidepressant | 93413-69-5 | C17H27NO2 | 277.2 | 9.3 | 3.28 |

Table S1. Physicochemical properties and structural features of the 11 target compounds analysed

Fig. S1 Flowsheet of the system studied. Green and red dotted lines represent the system sub-systems boundaries.

Table S2. Life Cycle Inventory of Sterilized Grain production. All values are referred to 1 kg of sterilized grain.

| **INPUT** |  |  |
| --- | --- | --- |
| *Materials/fuels* |  |  |
| Wheat grain, organic {GLO}\| market for wheat grain \| Conseq, U | 0.73 | kg |
| Tap water {Europe without Switzerland} \| tapwater production, underground water without treatment \| Conseq, U | 10.14 | kg |
| Calcium Sulfate | 2.17 | g |
| *Electricity/heat* |  |  |
| Electricity, low voltage {SE}\| market for\| Conseq, U | 1.2 | kWh |
| **OUTPUT** |  |  |
| Sterilized Grain | 1 | kg |
| *Waste to treatment* |  |  |
| Wastewater, average {Europe without Switzerland} \| market for wastewater \| Conseq, U | 9.87 | kg |

Table S3. Life Cycle Inventory of Liquid Culture production. All values are referred to 1L of liquid culture produced.

| **INPUT** |  |  |
| --- | --- | --- |
| *Materials/fuels* |  |  |
| Water ultrapure {RER}\| market for water, ultrapure \| Conseq, U | 0.99 | L |
| Sugar from sugar beet {ROW}\| beet sugar production, \| Conseq, U | 38.3 | g |
| Isopropanol {RER}\| isopropanol production, […] \| Conseq, U | 3.3 | g |
| Nylon 6 {RER}\|, market for nylon 6 \| Conseq, U | 2.2 | g |
| Aluminum, primary, liquid {UN-EUROPE} aluminium production, […] \| Conseq, U | 1.7 | g |
| Tissue paper {RER}\| tissue paper production, […] \| Conseq, U | 8.3 | g |
| *Electricity/heat* |  |  |
| Electricity, low voltage {SE}\| market for\| Conseq, U | 0.7 | kWh |
| **OUTPUT** |  |  |
| Liquid Culture | 1 | L |
| *Waste to treatment* |  |  |
| Aluminum (waste treatment) {GLO}\| recycling of aluminum \| Conseq, U | 1.66 | g |
| Waste Incineration of plastic (Nylon 6, Nylon 66, PAN), EU-27 | 2.2 | g |
| Wastepaper, sorted (waste treatment) {RER} \| tissue paper production \| Conseq, U | 11.6 | g |

Table S4. Life Cycle Inventory of White-Root-Fungi pellet based biological (WRF) treatment. All values are referred to F.U. (1 L of simulated wastewater)

| **INPUT** |  |  |
| --- | --- | --- |
| *Materials/fuels* |  |  |
| Residual Softwood, wet {ROW}\| market for residual softwood, wet \| Conseq, U (lignocellulosic support) | 5 | g |
| Grain Spawn | 40 | g |
| Silca Sand,{ROW}\| market for silica sand \| Conseq, U | 0.14 | kg |
| Simulated Spiked Wastewater | 1 | L |
| *Electricity/heat* |  |  |
| Electricity, low voltage {SE}\| market for\| Conseq, U (growth of WRF and maintenance) | 0.0146 | kWh |
| **OUTPUT** |  |  |
| Treated Simulated Spiked Wastewater | 1 | L |
| *Waste to treatment – Scenario 1* |  |  |
| Spent Biomass Landfill | 45 | g |
| Re-Use of backwashed sand | 0.14 | kg |
| Backwashing wastewater treatment | - |  |
| *Waste to treatment – Scenario 2* |  |  |
| Spent Biomass Incineration | 45 | g |
| Re-use of backwashed sand | 0.14 | kg |
| Backwashing wastewater treatment | - |  |

Table S5. Assumptions made for the Life Cycle Inventory developed for the preparation of 1 kg of wheat grain. (Ref. Table S2)

1. The wheat grain preparation process was adapted from Jarial et al. ^1^
2. Wheat grain (12% w/w moisture content) was assumed as precursor for grain spawn production
3. Tap water was used to wash, rinse, soak, boil, and sterilize the wheat grain at a weight ratio of 1: 5:3:3:2 (w/w)
4. 0.3% w_gypsum_/w_grain_ of gypsum (calcium sulfate) was assumed to be added to the grain during boiling to maintain a proper pH and prevent grain sticking
5. It was assumed that final sterilized wheat grain has 50% w/w moisture content
6. The heat required for boiling the wheat grain was calculated based on the enthalpy change required to rise the water from room temperature 25 $℃ (T_{1})$ 1.0 bar, to 100 ℃ ($T_{2})$, assuming at specific heat of water of 4.186 kJ/kg/°C, according to Eq.S1):

$\Delta H={(m}_{water}{\times cp}_{water}) \times( T_{2}-T_{1})$ )	Eq.S1)

This yield, considering 2 kg of water, 627.2 kJ.

7. The heat required for boiling wheat grain sterilization was calculated by applying Eq.S1, as the sum of the enthalpy change required (i) for increasing the water temperature at the water boiling point at 22 psi (1.5 bar), which is 112 ℃, and (ii) for vaporizing the water, considering 2182 kJ/kg as the evaporation enthalpy of water at 1.5 bar, which gives a total amount of 4363.4 kJ. For simplicity, the heat losses were considered to be negligible.
8. The total amount of energy required for grain preparation was estimated as the sum of the heat required for boiling the wheat grain (627.2 kJ) and to sterilize them (5091 kJ), which gives 5718.2 kJ (1.6 kWh) per kg wheat grain.
9. The wastewater generated from the rinse, soak, boiling and sterilizing phases of wheat grain is assumed to be sent for treatment by ordinary municipal collectors.

Table S6. Assumptions made for the Life Cycle Inventory of the production of 1 L of Liquid culture. (Ref. Table S3)

1. 1 L of Ultrapure (UP water) water was used to prepare the liquid culture
2. 4% w_UPwater_/w_sugar_, from sugar beet, was used as the source of glucose for mushroom myceli
3. Isopropyl alcohol was used as a cleaning agent to maintain an aseptic canning jar, and the syringe and airport lid were used for liquid culture production
4. Single-use tissue paper was used for isopropyl alcohol. After use they are sent for waste treatment
5. Aluminum foil was used to cover the airport lid of the jar during sterilization. After use, the aluminum foil was assumed to be recycled.
6. Liquid culture sterilization was performed in a pressure cocker under the same operational conditions used for wheat grain at point 7 in Table S3.
7. The amount of electricity required to sterilize the liquid culture was calculated following the same criteria of enthalpy change described at point 7 in Table S1, considering an initial amount of water of 1 kg, which gives a total value of 1560 kJ (0.4 kWh)
8. A single-use nylon syringe (2.2 g/item) was used as packaging for mushroom mycelium. After inoculation, nylon syringe was assumed to be sent for incineration
9. The preparation of mushroom mycelium inoculum is kept out of the scope of this study
10. The liquid culture was incubated at room temperature in the dark, which did not imply any additional indirect environmental impact
11. Grain spawn production was assumed to be performed by inoculating 4.4 mL of incubated liquid culture in 1 kg of sterilized wheat grain, which was colonized before use at room temperature.

Table S7. Assumptions made for the Life Cycle Inventory of the WRF growth and successive WFR WW treatment. (Ref. Table S4)

1. For the WW treatment 40 g/l of grain spawn prepared as reported in Tables S1-2 used and 5 g of lignin were used. The indirect environmental impact associated to lignin production was considered equal to that associated with the production of residual softwood, trough the ecoinvent process (residual softwood, wet {ROW}| market for residual softwood, wet | Conseq, U)
2. The simulated spiked WW was modeled according to the initial and final concentrations of the pharmaceuticals measured during the laboratory assay, as reported in Table S9
3. The electric energy consumption associated with air injection and mixing during the growth phase of WFR pellets and their maintenance was calculated assuming the use of a 35 W air pump capable of providing 40 L/min of air (8L/min of O_2_). Considering an O_2_ consumption of 2.05 g/g _biomass_.day during 4 days of growth, and 0.1 g/g biomass. per day, assuming 1 day maintenance, the total amount of energy required was 0.01458 KWh per L WW
4. For the sand filtration step, the scale factor of a pilot-scale sand filtration unit ^2^ 0.14 kg silica sand was considered. The silica sand is assumed to be reused in the same system after backwashing, and the amount of water used for backwashing is considered negligible at this stage.

Table S8. Concentration of selected pharmaceuticals in experiment 1 and 2. Mean ± std is shown, n=3

| Pharmaceuticals | Experiment 1 |  | Experiment 2 |  |  |  |
| --- | --- | --- | --- | --- | --- | --- |
|  | Initial Conc (µg/L) | Final Conc (µg/L) | Initial conc. (ng/L) | Conc. 2 h (ng/L) | Conc. 6 h (ng/l) | Conc. 24 h (ng/L) |
| Azithromycin | 10.2±0.8a* | 2.5±0.2b | BDL** | BDL | BDL | BDL |
| Erythromycin | 8.8±0.4a | 4.3±0.2b | 5.6±1.7a | 6.7±0.3a | 5.2±0.8a | 4.9±0.9a |
| Sulfamethoxazole | 5.9±0.6a | 0.8±0.1b | 676.7±20.8a | 333.3±66.3b | 210.0±96.4b | 216.7±32.1b |
| Caffeine | 10.1±0.8a | 8.0±0.4a | 52.7±5.9a | 36.0±8.7ab | 29.0±9.5ab | 26.7±11.9b |
| Fluconazole | 8.1±0.2a | 8.6±0.5a | 60.0±5.6a | 75.7±18.3a | 65.3±4.9a | 62.0±4.6a |
| Tramadol | 24±1.0a | 18.3±1.5b | 273.3±15.3a | 119.3±21.0b | 62.7±2.1c | 53.3±4.9c |
| Trimethoprim | 11.3±0.6a | 4.1±0.4b | 85.3±4.0a | 40.3±3.5b | 26.7±3.1c | 17.3±0.6d |
| Metoprolol | 16.3±0.6a | 10.6±1.2b | 118.7±22.0a | 102.3±15.3b | 74.7±5.8bc | 58.7±4.2c |
| Lidocaine | 15.7±0.6a | 11.7±0.6b | 86.0±6.1a | 76.0±22.1a | 43.0±6.6b | 37.0±5.0b |
| Venlafaxine | 12.0±1.0a | 7.3±0.3b | 416.7±32.1a | 230±17.3b | 160.0±34.6c | 126.7±11.6c |
| Desvenlafaxine | 12.3±0.6a | 9.5±0.6b | 1833.0±252.0a | 740.0±96.4b | 853±300b | 636.7±140.1b |

*Values followed by different letters, within each experiment, indicate significant difference (p<0.05), ** Below detection limit
